# Association between 2D landing biomechanics, isokinetic muscle strength and asymmetry in females using novel, task specific metrics based on ACL injury mechanisms

**DOI:** 10.1371/journal.pone.0326882

**Published:** 2025-07-01

**Authors:** Chelsea Oxendale, Grace Smith

**Affiliations:** 1 School of Sport and Exercise Sciences, Liverpool John Moores University, Liverpool, United Kingdom; 2 Division of Public Health, Sport and Wellbeing, University of Chester, Chester, United Kingdom; Universidade de Aveiro Escola Superior de Saude de Aveiro, PORTUGAL

## Abstract

This study investigated the relationship between isokinetic muscle strength metrics, landing biomechanics, and their asymmetries, in females. Twenty-three female team sport athletes completed unilateral forward drop landings, and isokinetic muscle strength assessment of the knee extensors and flexors, on both limbs. Discrete two-dimensional kinematics of the trunk, hip, knee, and ankle in the sagittal and frontal plane and peak GRF were recorded during the drop landings. Novel, task-specific isokinetic strength metrics related to the landing task, such as peak concentric and eccentric torque, angle specific torque (AST), functional range and traditional/functional ratios were quantified. Asymmetry for kinematic and muscle strength data were quantified based on the individual variability of the task and the population mean and smallest worthwhile change. Functional concentric flexor range explained 15–18% of the variance in peak frontal trunk (*P* = 0.003) and hip motion (*P* = 0.007) and 22% in peak frontal knee motion (*P* = 0.005), when combined with the functional flexion ratio. Peak eccentric extensor torque explained 13–14% of the variance in peak sagittal hip (*P* = 0.014) and knee (*P* = 0.009) motion. Asymmetry in concentric extensor AST explained 28% of the variance in peak knee frontal plane asymmetry (*P* = 0.010), however the direction of asymmetry was rarely present on the same side for kinematic and strength variables. Novel and task specific isokinetic strength metrics explained small but significant variances in sagittal and frontal plane landing kinematics and asymmetry, which have previously been related to ACL injury risk.

## Introduction

Anterior Cruiciate Ligament (ACL) injuries are amongst the most burdensome injuries in female team sports [1], resulting in high rates of hospital admission [2] and requiring a median recovery time of 10 months [1]. The majority of ACL injuries in female team sports are non-contact in nature (53–87%), and often occur during a landing, cutting or deceleration movement [3], with landing being the most reported mechanism [3,4]. Within the same sport, females are ~ 2–3-fold more likely to sustain an ACL injury compared with males, and non-contact ACL injuries are higher in amateur compared with elite-level female athletes (0.27 *vs.* 0.10 per 1000 player-hours) [5]. Lower limb strength and landing biomechanics have been prospectively linked with non-contact ACL injury risk in female athletes [6], and improvements in these factors can reduce injury risk [7]. Accordingly, further exploration of the association between lower limb strength and landing biomechanics in amateur female athletes may help inform more effective injury prevention strategies in this cohort.

Previous literature has explored the biomechanical mechanisms of non-contact ACL injuries in female athletes, using multiple 2D camera angles at the time of injury [4,8]. Knee abduction was the most common mechanism observed (75–88%), often accompanied by trunk lateral flexion toward the injured limb, hip adduction, and a knee-dominant landing strategy characterized by limited flexion at the trunk, hip, and ankle [9]. Prospectively, greater lateral flexion of the trunk [10,11], knee abduction [10,11] and vertical GRF [11] during a landing task, for example, can differentiate between athletes who are at greater risk of sustaining an ACL injury. Research has therefore employed single leg landings off a raised platform [12,13] to examine the biomechanics of landing as a screening method for lower limb injuries, which offer greater insights into ACL injury risk factors compared with double leg landings [14]. Such screening methods have demonstrated some ability to identify high risk athletes [15], and demonstrate similar landing biomechanics to a forward vertical jump [16]. The assessment of sagittal and frontal landing kinematics with 2D motion capture also offers a field friendly assessment of injury risk, which has shown good to excellent agreement with 3D motion capture [17].

Strength of the quadriceps and hamstrings have been prospectively linked to ACL injury risk [18], as these muscles are the main antagonists (i.e., loaders) and agonists (i.e., supporters) of the ACL during landing tasks [19]. For example, the hamstring-to-quadricep ratio is related to greater knee loading during a landing task [13] and an increased risk of ACL injury [18]. Whilst conflicting evidence suggests the hamstring-to-quadricep ratio is not an independent risk factor of ACL injuries [20], the angular velocities typically employed (60°^.^s^−1^) do not mimic those observed during forward landings tasks (188.9 ± 220.6°^.^s^−1^ at peak vertical GRF) [21]. The assessment of eccentric torque, angular specific torque, functional torque range (the angular range at which a percentage of peak torque is maintained) and functional ratios have also been recommended for injury screening in team sport athletes [20,22,23]. For example, the quadriceps primarily perform eccentric work and the hamstring perform concentric work during the initial phase of a landing [24], which can be quantified as a functional flexion ratio. Assessment of the functional flexion ratio [25], torque in more extended knee positions [20,23] and functional torque range [23] may better reflect the movement patterns/muscle actions commonly observed during landing actions, when ACL injuries typically occur.

Limb preference, referred to as the preferred limb for a given motor task, and limb asymmetry can also influence ACL injury risk in females. Specifically, female athletes are more likely to injury their left/ non-preferred ACL compared with the right/ preferred limb [26] which has been partly attributed to kinematic and strength asymmetries between limbs. For example, reduced sagittal plane range of motion in the hip and knee joints has been observed in the non-preferred compared with the preferred leg [27] and quadricep strength asymmetries of >15% increase peak vertical GRF during landing in the ACL reconstructed limb compared with the non-injured limb [28]. Whilst these reports identify the potential mechanisms of limb asymmetry on ACL injury risk, the use of arbitrary asymmetry thresholds (e.g., > 10–15%) has been critiqued [29] in favour of an individualised approach to asymmetry. Briefly, considering the individual variability of a task (CV%) as well as the population mean and smallest worthwhile change, can help determine meaningful asymmetry in the context of the specific population and metric assessed [30]. Literature assessing asymmetries in females has also focused on an injured population, comparing the injured and non-injured limb to inform rehabilitation [31]. Assessment of kinematic and strength asymmetries and their association in uninjured female athletes might better inform preventative injury programmes.

Whilst the literature highlights the importance of landing kinematics, muscular strength and asymmetry, these factors have typically been examined in isolation. Greater understanding of the interaction between risk factors is needed to develop a comprehensive athlete risk profile and plan effective prevention interventions [32]. The aims of the present study were twofold. Firstly, to identify the extent to which novel measurements of isokinetic muscle strength explain variance in landing biomechanics and secondly, to quantify muscle strength asymmetries, landing biomechanics asymmetries and the variance they explain. It was hypothesized that novel isokinetic variables, more relevant to the landing task (i.e., functional ratios, angle specific torques and functional range), will explain some of the variance in single leg landing biomechanics, more so than traditional isokinetic variables (e.g., hamstring-to-quadricep ratio).

## Methods

### Subjects

A total of 23 females (age: 22.2 ± 3.8 years; stature: 1.67 ± 0.1 m; mass: 65.6 ± 6.6 kg) participated in the study. An *a prior* power analysis determined a minimum sample size of 19 was required to detect a moderate two-tailed correlation (*r* = 0.6) with 80% power at a type I error of 0.05, based on reported associations between quadricep strength and knee kinematics (*r* = 0.64) [33]. All subjects took part in a competitive team sport at least once per week (netball, football, rugby, hockey, or volleyball) and had at least 6 months previous playing experience. Subjects also had no previous history of knee surgery and/or no lower limb injuries in the past 6 months. The subject’s preferred leg was determined by asking which leg they would use to kick a ball [34,35]. All subjects provided written informed consent to take part in the study. This project received ethical approval from the University of Chester Faculty of Medicine and Life Sciences Research Ethics Committee (1849-22-CO-SES), and all procedures were conducted according to the Declaration of Helsinki. The start and end of the recruitment period for this study was from 06/05/2022–06/04/2023.

### Design

In a repeated measures design, subjects performed unilateral forward drop landings on both limbs. Thereafter, an assessment of isokinetic muscle strength of the knee extensors and flexors was taken on both limbs. All testing was conducted in one visit.

### Landing biomechanics

Eighteen reflective markers were attached onto the subject’s greater tuberosity of the humeral head, trochanter major, anterior superior iliac spine, medial and lateral femoral condyles, distal tibia, medial and lateral malleolus, and 5th metatarsal head on both sides of the body. Two markers were placed at the midpoint of the lateral and medial femoral condyles on the patella. Subjects then performed a standardised warm-up consisting of several double-leg squats and countermovement jumps and familiarized themselves with the landing task by performing 3 practice repetitions on both limbs.

Subjects then performed 7 unilateral forward drop landings alternating between the right and left leg [17]. Subjects began standing unilaterally on a 30 cm high box, dropped forward to a target half of their body height on the same leg, maintaining balance on one foot for 2 s for the trial to be successful [17]. A trial was deemed unsuccessful if the subject’s non-support leg touched the ground, or if they lost balance during the test. The middle five successful trials were used for analysis [36,37]. During each landing sagittal and frontal plane movements were simultaneously captured with two digital video cameras (Quintic high speed camera GigE Live, Quintic, United Kingdom) sampling at 100 fps, positioned ~ 3 m to the front and side of the landing zone at a height of ~1 m. GRF data were captured using one embedded force platform (Kistler, 9281 CA, Switzerland), sampling at 1200 Hz.

### Isokinetic muscle strength

Isokinetic knee extensor and flexor concentric and eccentric peak torque at 180°^.^s^−1^ was assessed using a dynamometer (Biodex Medical, System 4, New York, USA) on both limbs. Prior to testing, all subjects performed a submaximal isokinetic warm up consisting of 10 extension-flexion repetitions at 120°^.^s^−1^. Thereafter, subjects performed 5 maximal concentric extension and flexion efforts at 180°^.^s^−1^ for familiarization and after a 2-minute rest period, performed 5 maximal concentric extension and flexion efforts at 180°^.^s^−1^ [23], across an approximate range of motion of 90–100 degrees. The familiarization and assessment of 5 maximal eccentric extension and flexion efforts then took place in the same format, across an approximate range of motion of 85–95 degrees, following a warmup of 5 submaximal eccentric repetitions at 120°^.^s^−1^. The eccentric torque limit for flexion and extension was set to 2.5 and 2 multiples of peak concentric torque, based on pilot testing, respectively. Subjects were verbally encouraged throughout and instructed to extend (“kick out”) and flex (“pull back”) as hard and as fast as possible for concentric efforts, and resist/stop the movement as much as possible for eccentric efforts. The average range of motion for all participants for concentric and eccentric efforts was 95.1 ± 8.2° and 87.8 ± 8.2°, respectively.

### 2D video and GRF data processing

For the 2D video analysis, reflective markers were automatically digitised using Quintic software (Quintic Biomechanics v31, Quintic consultancy limited, UK). In the sagittal plane, trunk flexion (relative to vertical), hip flexion, knee flexion and ankle dorsiflexion angles were calculated. In the frontal plane, knee frontal plane projection angle (FPPA) was calculated using the marker on the patella for the knee joint centre, the ASIS markers and the distal tibia, then subtracted from 180° and hip adduction was defined as the angle between the bilateral ASIS and the patella, then subtracted from 90° [38]. Lateral trunk flexion was calculated as the perpendicular line from the ipsilateral ASIS to the midpoint between the two humeral head markers [38].

Data was smoothed using a fourth-order Butterworth low pass filter and Quintic’s recommended cut off frequencies of 12–24 Hz based on residual analysis of each marker trajectory. Discrete peak variables were quantified over the weight acceptance phase of the landing, defined as initial contact to peak knee flexion [39]. Vertical GRF was normalised to subjects’ body weight, filtered with a Butterworth low pass filter with a cut off frequency of 100 Hz.

### Isokinetic muscle strength data processing

Torque data was smoothed using a fourth-order Butterworth low pass filter with a 5 Hz cut-off frequency. The isokinetic phase of each repetition within 5% of the predetermined constant angular velocity (180°^.^s^−1^) was identified and the repetition eliciting the highest peak concentric and eccentric extensor and flexor torque was used for further analysis. Peak concentric angular specific torque (AST) at 40° of knee flexion, and the functional range where 85% of peak torque was maintained in a single repetition [23], were calculated and expressed relative to the subject’s body mass. The maximal concentric and eccentric hamstring-to-quadricep ratio was calculated by dividing the peak knee flexor torque by the peak knee extensor torque for each contraction type. A functional flexion ratio was also calculated as peak concentric flexor torque divided by peak eccentric extensor torque [25]. Measures of concentric and eccentric peak torque, AST and functional ratios have previously displayed favourable reliability (ICC > 0.9) at an angular velocity of 60°^.^s^−1^ [40].

### Statistical analysis

Descriptive statistics (mean ± standard deviation) for all isokinetic muscle strength and landing biomechanics variables were calculated. The coefficient of variance was calculated to assess isokinetic muscle strength intra-individual variability over two repetitions [41], the peak and second peak repetition, and kinematic/kinetic intra-individual variability over the 5 landing trials. Group analysis of kinematic, kinetic and strength asymmetry were calculated using the following equation:


Asymmetry index (absolute) = [(A – B) / (Max A, B)] x 100 

Were A is the value of interest on the preferred leg and B is the non-preferred leg [29].

Individual asymmetry was also assessed and only those with asymmetry indexes greater than both the intra-limb variability (using the highest CV between the preferred/non-preferred leg) and the threshold for moderate asymmetry (calculated as the group mean plus the smallest worthwhile change [SWC; 0.2 * between-subject SD]), were considered to have meaningful asymmetry [29,30]. Data on intra-limb variability are provided in S1 and S2 Tables.

Pearson correlation coefficients and 95% confidence intervals (using the Fisher Z transformation), with Bonferroni-adjusted *P* values were calculated to assist in the initial screening of isokinetic muscle strength predictor variables, by selecting correlations of *R* > 0.3. Both the preferred and non-preferred legs were included in the correlation yielding a sample of n = 46. All isokinetic muscle strength variables, excluding peak concentric extensor and flexor torque, which were strongly associated with angle specific torque (*R* ≥ 0.9), were included in the initial screening, yielding 9 pairwise correlations per kinematic variable. Similarly, associations between strength asymmetry with kinematic asymmetry (n = 23) were assessed using Spearman’s correlation coefficients and 95% confidence intervals, with Bonferroni-adjusted *P* values. Multiple backwards regression models (n = 4) were applied using selected predictor variables to explain the variance in kinematic response variables; peak trunk lateral flexion, peak knee FPPA and flexion angle and peak hip adduction angle and corresponding *P* values were Bonferroni-adjusted. One additional regression model was applied between selected predictor variables and peak knee FPPA asymmetry. Only muscle strength variables with *R* > 0.3 were used as potential predictor variables in the regression models, to reduce potential overfitting [42]. The *F* probability for variable entry was set at 0.05 and for variable removal was set at 0.10. Assumptions of the regression models were met. The alpha level for statistical significance was set at 0.05 level.

## Results

### Isokinetic muscle strength

Muscle function data on the preferred and non-preferred legs are presented in Table 1. A total of 22 out of 23 subjects indicated they preferred the right side and mean asymmetry ranged from 6–16% across isokinetic muscle strength variables. Individual isokinetic muscle strength data are available in S3 File.

**Table 1:** Measures of muscle strength on the preferred and non-preferred leg and asymmetry.

	Preferred leg	Non-preferred leg	Average Asymmetry (%)	Individuals with meaningful asymmetry (% [n])
Peak concentric extensor torque (N^.^m^.^kg^−1^)	1.65 ± 0.30	1.58 ± 0.24	6.5 ± 5.3	26 [n = 6]
Functional concentric extensor range (°)	42.0 ± 6.2	42.3 ± 6.2	11.9 ± 10.6	30 [n = 7]
Angle specific concentric extensor torque (N^.^m^.^kg^−1^)	1.35 ± 0.27	1.30 ± 0.24	10.1 ± 8.4	35 [n = 8]
Peak concentric flexor torque (N^.^m^.^kg^−1^)	0.87 ± 0.23	0.83 ± 0.17	9.3 ± 7.7	26 [n = 6]
Functional concentric flexor range (°)	47.9 ± 8.4	52.5 ± 7.7	16.1 ± 12.4	35 [n = 8]
Angle specific concentric flexor torque (N^.^m^.^kg^−1^)	0.82 ± 0.21	0.77 ± 0.14	11.2 ± 8.3	30 [n = 7]
Peak eccentric extensor torque (N^.^m^.^kg^−1^)	2.65 ± 0.65	2.57 ± 0.69	9.2 ± 9.1	22 [n = 5]
Peak eccentric flexor torque (N^.^m^.^kg^−1^)	1.84 ± 0.46	1.65 ± 0.39	13.8 ± 12.7	30 [n = 7]
Peak concentric hamstring to quadricep ratio (%)	52.4 ± 8.8	52.8 ± 8.7	8.9 ± 6.9	44 [n = 10]
Peak eccentric hamstring to quadricep ratio (%)	71.0 ± 14.3	67.6 ± 19.6	16.0 ± 14.0	35 [n = 8]
Functional flexion ratio (%)	34.3 ± 11.2	34.3 ± 11.0	14.5 ± 9.4	30 [n = 7]

### Landing biomechanics

Table 2 demonstrates landing biomechanics measures for the preferred and non-preferred legs. Mean asymmetry (%) ranged from <5% for knee flexion to >20% for peak knee abduction, hip adduction and trunk lateral flexion. Individual landing biomechanics data are available in S3 File.

**Table 2:** Landing kinematics and kinetics for the preferred and non-preferred leg and asymmetry.

	Preferred leg	Non-preferred leg	Mean Asymmetry (%)	Individuals with meaningful Asymmetry (% [n])
Peak knee flexion (°)	115.04 ± 10.71	116.33 ± 11.54	4.77 ± 3.53	30 [n = 7]
Peak knee FPPA abduction (°)	18.34 ± 7.73	17.53 ± 5.39	22.51 ± 12.89	39 [n = 9]
Peak hip flexion (°)	127.42 ± 14.74	126.91 ± 15.77	5.73 ± 4.40	39 [n = 9]
Peak hip adduction (°)	18.48 ± 6.36	15.57 ± 4.52	21.30 ± 18.23	39 [n = 9]
Peak ankle dorsiflexion (°)	92.92 ± 6.81	95.78 ± 8.43	4.83 ± 3.84	35 [n = 8]
Peak trunk lateral flexion (°)	13.84 ± 4.02	12.26 ± 3.00	24.88 ± 14.86	30 [n = 7]
Peak GRF (BW)	4.17 ± 0.56	4.23 ± 0.72	7.46 ± 6.23	13 [n = 3]

Functional concentric flexor range, functional flexion ratio, peak eccentric extensor torque and concentric extensor AST were the only strength variables to display correlations of *R* > 0.3 with landing kinematic variables (S1 File). These variables were incorporated into the relevant regression model. Functional concentric flexor range and functional flexion ratio explained 22% of the variance in peak knee FPPA and functional concentric flexor range explained 15–18% of the variance in peak frontal plane trunk and hip motion (Table 3). Peak eccentric extensor torque explained 14% of the variance in sagittal knee flexion angle.

**Table 3:** Multiple regression model summary of the association between independent variables with peak frontal trunk, hip, and knee angle and peak hip and knee flexion angle.

	*R* ^2^	Adjusted *R*^2^	*F* Statistic	*P* Value
**Peak knee frontal plane projection angle** Functional concentric flexor range Functional flexion ratio	0.217	0.180	5.950	0.020
**Peak hip adduction angle** Functional concentric flexor range	0.153	0.133	7.929	0.028
**Peak lateral trunk flexion** Functional concentric flexor range	0.183	0.164	9.853	0.012
**Peak knee flexion angle** Peak eccentric extensor torque	0.144	0.125	7.431	0.036

Peak concentric extensor AST asymmetry and peak eccentric flexor torque asymmetry were the only strength asymmetry variables to display correlations of *R* > 0.3 with peak knee FPPA asymmetry (S2 File). Asymmetry in concentric extensor AST explained 28% of the variance in peak knee frontal plane projection angle asymmetry (Table 4).

**Table 4:** Multiple regression model summary of the association between independent asymmetry variables with peak knee frontal plane projection angle asymmetry.

	*R* ^2^	Adjusted *R*^2^	*F* Statistic	*P* Value
**Peak knee frontal plane projection angle asymmetry** Peak concentric extensor AST asymmetry	0.276	0.241	7.989	0.010

**Fig 1:**
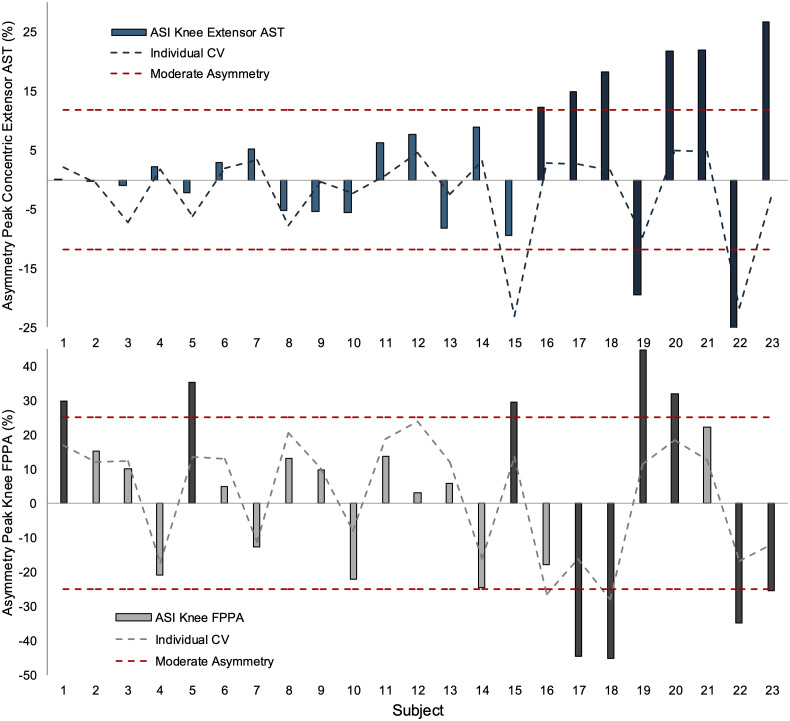
Peak knee concentric extensor AST asymmetry and peak knee FPPA asymmetry for individual subjects. ASI = asymmetry index. Individual asymmetry is determined based on intra-individual variability (dashed line) and moderate asymmetry. Darker bars are those displaying asymmetry. Positive asymmetry represents the preferred leg exhibited higher values than non-preferred leg, thus negative asymmetry depicts higher values on non-preferred leg. *Note: Subjects have been ordered from lowest peak extensor AST asymmetry (subject 1) to highest peak extensor AST asymmetry (subject 23) for illustrative purposes.*

Individual asymmetry for knee concentric extensor AST and knee FPPA are presented in Fig 1. Six of the eight subjects displaying meaningful asymmetry in knee extensor AST also displayed meaningful asymmetry in knee FPPA. Of these six subjects, only three demonstrated a stronger leg (positive asymmetry for knee extensor AST) combined with reduced knee FPPA (negative asymmetry) showing the direction of asymmetry was highly individual and task specific.

## Discussion

This study aimed to assess the relationship between isokinetic muscle strength metrics, landing biomechanics, and their asymmetries. In line with the first aim of the study, functional concentric flexor range and functional flexion ratio were the main isokinetic muscle strength variables to explain small (22%) but significant variance in knee frontal plane motion. Reported associations between hamstring muscle activity and knee FPPA have previously been varied, with one study demonstrating a significant relationship [43] and another finding no relationship [44]. The hamstrings act to unload the ACL when the knee is flexed more than ~20° [45] and ACL strain is significantly correlated to knee abduction angles [10]. Consequently, it is somewhat expected that maintaining higher strength through a range of knee flexion and high eccentric hamstring strength relative to concentric quadricep strength, explains some variance in knee FPPA. This also aligns with a higher ACL shear force reported during a landing task in females with lower peak knee flexor peak torque [13]. The functional concentric flexor range also explained some variance (15–18%) in frontal plane hip and trunk motion, which is consistent with the weak associations between lower extremity strength and trunk frontal motion reported in young athletes following ACL reconstruction [46]. Muscle weakness in distal segments can increase centre of mass displacement and lead to increased trunk motion during balance tasks [47]. Thus, a reduced range of knee flexion, where participants are still able to maintain strength, might lead to increased frontal motion at the hip and trunk as they attempt to stabilize their body during the landing task. The small variance explained likely reflects the influence of other muscle strength, and kinematic factors, as decreased hip abductor, extensor and external rotator strength [48] and hip external rotation range of motion [49] have been reported to predispose athletes to knee abduction during single leg landings. Further analysis of the present data also revealed 49% of the variance in knee FPPA was explained by peak hip adduction angle and peak GRF (S3 Table). Collectively, these data support the assessment of a player’s ability to maintain knee flexor strength over a given range of motion (i.e., functional range) at high angular velocities for injury screening [23] and also highlights the need for a multifactorial approach.

Novel and task specific metrics also explained a small proportion of variance (14%) in sagittal knee motion, whilst other metrics (e.g., hamstring to quadricep ratio) did not. Specifically, peak eccentric extensor torque was the only isokinetic variable to explain some variance in peak knee flexion. Previous literature has shown associations between leg strength and sagittal landing kinematics are varied, with some demonstrating no relationship [12], and others displaying significant associations between peak knee extensor torque and peak knee flexion angle [33]. However, direct comparisons are difficult due to differences in the task performed, the population evaluated (healthy vs. ACL reconstruction) and assessment of muscle strength variables. Increasing knee flexion motion may reduce the risk of ACL injury in females [50], thus identification of isokinetic strength variables related to sagittal motion could help inform injury screening practices. However, the small variance explained suggest screening of isokinetic variables related to sagittal motion during a single leg landing task should not be based solely on knee extensor isokinetic metrics.

A novel aspect of the study (aim 2) was to assess individual asymmetry in landing kinematics and muscle strength in a healthy population. For most isokinetic strength and kinematic variables, approximately one third of the subjects displayed meaningful asymmetry. This is lower than recent reports that 69% of females displayed asymmetry in peak extensor torque [51], and likely reflects differences in asymmetry cut off. Whilst these data question the comparison of the injured limb to the contralateral limb in rehabilitation practice [51], asymmetry in peak knee extensor AST explained a quarter of the variance in peak knee FPPA, and still have clinical relevance. Indeed, associations between asymmetry in muscle strength and single leg landing variables have previously been reported [46,52] and symmetry in concentric knee extensor torque has been associated with higher maintenance of sports participation after ACL reconstruction [53]. Notably, individual peak knee FPPA and knee extensor AST asymmetry data demonstrated great differences between subjects, not only with the magnitude of asymmetry but also the direction, with asymmetries rarely being present on the same side for kinematic and strength variables. This is consistent with the poor agreement (kappa coefficient: −0.33–0.1) between isokinetic strength asymmetry and countermovement jump asymmetry in female football players, indicating that asymmetry in favour of one limb during one test would unlikely correspond to the same limb in another test [54]. Taken together, these data reinforce an individualised approach to reporting asymmetries is warranted [30]. Practitioners should note the prevalence of biomechanical asymmetries in female team sport athletes and include comparisons between limbs, as well as to baseline values (or normative values), to inform return to sport criteria.

It is important to acknowledge limitations of the study. Firstly, knee FPPA was calculated from automatic 2D analysis, using markers and angle definitions in line with previous studies [17,38]. Ideally 3D analysis would be used, although 2D automatic methods have shown good to excellent agreement with 3D motion capture in knee FPPA [17]. Trial to trial intra-limb variability of the kinematic data in the present study was also assessed using CV% and frontal plane kinematics demonstrated higher variability (S1 Table). However, the chosen approach for quantifying meaningful asymmetry incorporates this variance as well as the smallest worthwhile change, meaning this is a more conservative method for assessing moderate asymmetry. In addition, while the use of a 30 cm drop height is often used in research assessing landing biomechanics [17], this might impose varying demands on athletes of different statures and should be considered in future research. The assessment of peak torque at higher angular velocities and during eccentric muscle actions used in the present study can also be less favourable [55]. The between repetition intra-limb variability (CV%) for all isokinetic strength metrics were assessed (S2 Table), which has previously been used to inform reliability [41]. All isokinetic strength metrics demonstrated a CV% of less than 8.5%, which is below the typical increases in average concentric and eccentric peak torque (8.5–23.9%) observed after 6 weeks of resistance training in female football players [56]. Finally, the use of correlations to identify isokinetic muscle strength predictor variables, ensured a maximum of two predictor variables were included in each regression model. Including ten subjects per predictor variable has been recommended in previous literature [42], thus the number of subjects used in the present study is sufficient to address the study aims.

## Conclusion

Due to the lack of standardised isokinetic protocols, we advocate the assessment of novel and task specific isokinetic variables which better reflect sport specific actions. In particular, the assessment of functional concentric flexor range, functional flexor ratio and eccentric extensor peak torque at high angular velocities (180°^.^s^−1^). These variables explain some of the variance in frontal and sagittal plane motion during single leg landings, commonly associated with ACL injury risk. The small variance explained reaffirms the need for a multifactorial approach to injury screening, as kinematic and kinetic factors explained more variance in FPPA compared with isokinetic muscle strength. We also recommend an individualised approach for quantifying interlimb asymmetry when designing training programmes or assessing return to sport criteria, specifically the use of concentric extensor AST asymmetry, which explained a quarter of the variance in knee FPPA asymmetry.

## Supporting information

S1 File Relationship between isokinetic muscle strength variables and landing biomechanics. (DOCX)

S2 File Relationship between isokinetic muscle strength asymmetry and landing biomechanics asymmetry. (DOCX)

S3 File Isokinetic strength and landing biomechanics individual raw data. (XLSX)

S1 Table Average intra-individual variability of landing kinematics and kinetics for the preferred and non-preferred leg CV %. (DOCX)

S2 Table Average intra-individual variability of isokinetic strength metrics for the preferred and non-preferred leg CV %. (DOCX)

S3 Table Multiple regression model summary of the association between independent kinematic/kinetic variables with peak knee frontal plane projection angle. (DOCX)
