# Supplementary material for: Association between 2D landing biomechanics, isokinetic muscle strength and asymmetry in females using novel, task specific metrics based on ACL injury mechanisms
Source: PLoS One. 2025 Jul 1;20(7):e0326882. doi: 10.1371/journal.pone.0326882 (PMC12212501; doi:10.1371/journal.pone.0326882)
Supplement: S1 File — (DOCX) [file pone.0326882.s001.docx]

**Supplementary file S1 Relationship between isokinetic muscle strength variables and landing biomechanics**

Pearson’s correlation coefficients were calculated between isokinetic muscle strength variables and landing biomechanics, with 95% confidence intervals and Bonferroni-adjusted p-values. Functional concentric flexor range (*r* = -0.397 [-0.617 – -0.121], *P* = 0.054) and functional flexion ratio (*r* = -0.329 [-0.565 – -0.043], *P* = 0.230) demonstrated weak associations with peak knee FPPA. Functional concentric flexor range also demonstrated a weak relationship with peak hip adduction angle (*r* = -0.391 [-0.612 – -0.157], *P* = 0.063) and a moderate relationship with peak lateral trunk flexion (*r* = -0.428 [-0.639 – -0.122], *P* = 0.027). In the sagittal plane, peak eccentric extensor torque (*r* = 0.380 [0.101 – 0.604], *P* = 0.081]) and extensor concentric AST (*r* = 0.358 [0.075 – 0.587], *P* = 0.131) demonstrated weak associations with peak knee flexion. Finally, peak eccentric extensor torque (*r* = 0.359 [0.076 – 0.588], *P* = 0.126) displayed a weak association with peak hip flexion. No other correlations with *R* > 0.3 were evident.
