# Supplementary material for: Association between 2D landing biomechanics, isokinetic muscle strength and asymmetry in females using novel, task specific metrics based on ACL injury mechanisms
Source: PLoS One. 2025 Jul 1;20(7):e0326882. doi: 10.1371/journal.pone.0326882 (PMC12212501; doi:10.1371/journal.pone.0326882)
Supplement: S2 File — (DOCX) [file pone.0326882.s002.docx]

**Supplementary file S2: Relationship between isokinetic muscle strength asymmetry and landing biomechanics asymmetry.**

Spearman’s correlation coefficients were calculated to assess the relationship between isokinetic muscle strength asymmetry and landing biomechanics asymmetry, with 95% confidence intervals and Bonferroni-adjusted p-values. A Spearman’s correlation revealed a weak-to-moderate correlation between peak knee FPPA asymmetry with peak concentric extensor AST asymmetry (*r* = 0.447 [0.030 – 0.732], *P* = 0.297) and a weak relationship with peak eccentric flexor torque (*r* = -0.325 [-0.658 – 0.113], *P* = 1.0). No other correlations with *R* > 0.3 were evident.
