# Supplementary material for: Association between 2D landing biomechanics, isokinetic muscle strength and asymmetry in females using novel, task specific metrics based on ACL injury mechanisms
Source: PLoS One. 2025 Jul 1;20(7):e0326882. doi: 10.1371/journal.pone.0326882 (PMC12212501; doi:10.1371/journal.pone.0326882)
Supplement: S1 Table — (DOCX) [file pone.0326882.s004.docx]

**Table S1.** Average intra-individual variability of landing kinematics and kinetics for the preferred and non-preferred leg CV %

|  | **Preferred leg (%)** | **Non-preferred leg (%)** |
| --- | --- | --- |
| Peak knee flexion (°) | 3.9 | 3.4 |
| Peak knee FPPA abduction (°) | 12.1 | 12.9 |
|  |  |  |
| Peak hip flexion (°) | 3.5 | 4.2 |
| Peak hip adduction (°) | 13.2 | 16.0 |
| Peak ankle dorsiflexion (°) | 2.9 | 2.7 |
|  |  |  |
| Peak trunk lateral flexion (°) | 18.7 | 21.3 |
| Peak GRF (BW) | 6.2 | 6.7 |
|  |  |  |
