# Supplementary material for: Association between 2D landing biomechanics, isokinetic muscle strength and asymmetry in females using novel, task specific metrics based on ACL injury mechanisms
Source: PLoS One. 2025 Jul 1;20(7):e0326882. doi: 10.1371/journal.pone.0326882 (PMC12212501; doi:10.1371/journal.pone.0326882)
Supplement: S2 Table — (DOCX) [file pone.0326882.s005.docx]

**Table S2.** Average intra-individual variability of isokinetic strength metrics for the preferred and non-preferred leg CV %

|  | **Preferred leg (%)** | **Non-preferred leg (%)** |
| --- | --- | --- |
| Peak concentric extensor torque | 3.3 | 2.0 |
| Functional concentric extensor range | 8.2* | 8.0 |
| Angle specific concentric extensor torque | 3.3 | 3.5 |
| Peak concentric flexor torque | 2.4 | 2.3 |
| Functional concentric flexor range | 8.3* | 8.4 |
| Angle specific concentric flexor torque | 2.7 | 3.1 |
| Peak eccentric extensor torque | 5.9 | 6.8 |
| Peak eccentric flexor torque | 4.5* | 6.7 |
| Peak concentric hamstring to quadricep ratio | 2.1 | 1.8 |
| Peak eccentric hamstring to quadricep ratio | 5.1* | 6.1 |
| Functional flexion ratio | 5.5 | 5.0 |

** note that one participant failed to achieve a second repetition were torque exceeded 85% of peak torque or failed to achieve a second repetition at a constant angular velocity and was excluded from this analysis.*
