# Supplementary material for: Association between 2D landing biomechanics, isokinetic muscle strength and asymmetry in females using novel, task specific metrics based on ACL injury mechanisms
Source: PLoS One. 2025 Jul 1;20(7):e0326882. doi: 10.1371/journal.pone.0326882 (PMC12212501; doi:10.1371/journal.pone.0326882)
Supplement: S3 Table — (DOCX) [file pone.0326882.s006.docx]

**Table S3.** Multiple regression model summary of the association between independent kinematic/kinetic variables with peak knee frontal plane projection angle.

|  | R^2^ | Adjusted R^2^ | F Statistic | P Value |
| --- | --- | --- | --- | --- |
| **Peak knee frontal plane projection angle**   1. Peak hip adduction angle 2. Peak GRF (xBW) | 0.487 | 0.463 | 20.417 | <0.001 |
